# Blood Culture Use in Medical and Surgical Intensive Care Units and Wards

**DOI:** 10.1001/jamanetworkopen.2024.54738

**Published:** 2025-01-15

**Authors:** Valeria Fabre, Yea-Jen Hsu, Karen C. Carroll, Alejandra B. Salinas, Avinash Gadala, Chris Bower, Sarah Boyd, Kathleen O. Degnan, Pragya Dhaubhadel, Daniel J. Diekema, Marci Drees, Baevin Feeser, Mark A. Fisher, Cynthia Flynn, Bradley Ford, Erin B. Gettler, Laurel J. Glaser, Jessica Howard-Anderson, J. Kristie Johnson, Justin J. Kim, Marvin Martinez, Amy J. Mathers, Leonard A. Mermel, Rebekah W. Moehring, George E. Nelson, John C. O’Horo, Dana E. Pepe, Evan D. Robinson, Guillermo Rodríguez-Nava, Jonathan H. Ryder, Jorge L. Salinas, Gregory M. Schrank, Aditya Shah, Mark Shelly, Emily S. Spivak, Kathleen O. Stewart, Thomas R. Talbot, Trevor C. Van Schooneveld, Anastasia Wasylyshyn, Sara E. Cosgrove

**Affiliations:** 1 Department of Medicine, Division of Infectious Diseases, Johns Hopkins University School of Medicine, Baltimore, Maryland; 2 Department of Health Policy and Management, Johns Hopkins Bloomberg of School of Public Health, Baltimore, Maryland; 3 Department of Pathology, Division of Medical Microbiology, Johns Hopkins University School of Medicine, Baltimore, Maryland; 4 Department of Hospital Epidemiology and Infection Control, The Johns Hopkins Hospital, Baltimore, Maryland; 5 Department of Medicine, Division of Infectious Diseases, Emory University School of Medicine, Atlanta, Georgia; 6 Saint Luke’s Health System, Kansas City, Missouri; 7 Department of Medicine, Division of Infectious Diseases, University of Pennsylvania, Philadelphia; 8 Geisinger Medical Center, Danville, Pennsylvania; 9 Department of Medicine, Division of Infectious Diseases, University of Iowa Hospitals and Clinics, Iowa City; 10 ChristianaCare, Wilmington, Delaware; 11 Division of Infection Control/Hospital Epidemiology, Beth Israel Deaconess Medical Center, Boston, Massachusetts; 12 Department of Pathology, University of Utah School of Medicine, Salt Lake City; 13 Department of Pathology, University of Iowa Carver College of Medicine and University of Iowa Hospitals and Clinics, Iowa City; 14 Division of Infectious Diseases, Duke University, Durham, North Carolina; 15 Department of Pathology and Laboratory Medicine, University of Pennsylvania, Philadelphia; 16 Department of Epidemiology, University of Maryland School of Medicine, Baltimore; 17 Department of Medicine, Dartmouth Hitchcock Medical Center, Lebanon, New Hampshire; 18 Lifespan Hospital System, Providence, Rhode Island; 19 Division of Infectious Diseases and International Health, Department of Medicine, University of Virginia Health, Charlottesville; 20 Warren Alpert Medical School of Brown University, Providence, Rhode Island; 21 Department of Medicine, Division of Infectious Diseases, Vanderbilt University School of Medicine, Nashville, Tennessee; 22 Division of Public Health, Infectious Diseases and Occupational Medicine, Mayo Clinic, Rochester, Minnesota; 23 Department of Medicine, Division of Infectious Diseases, Beth Israel Deaconess Medical Center, Boston, Massachusetts; 24 Division of Infectious Diseases, Stanford University School of Medicine, Stanford, California; 25 Department of Internal Medicine, Division of Infectious Diseases, University of Nebraska Medical Center, Omaha; 26 Department of Medicine, University of Maryland School of Medicine, Baltimore; 27 Division of Infectious Diseases, Department of Internal Medicine, University of Utah School of Medicine, Salt Lake City; 28 Department of Quality Assurance and Safety, Dartmouth Hitchcock Medical Center, Lebanon, New Hampshire; 29 Department of Medicine, Division of Infectious Disease, University of Michigan Health, Ann Arbor

## Abstract

**Importance:**

Blood culture (BC) use benchmarks in US hospitals have not been defined.

**Objective:**

To characterize BC use in adult intensive care units (ICUs) and wards in US hospitals.

**Design, Setting, and Participants:**

A retrospective cross-sectional study of BC use in adult medical ICUs, medical-surgical ICUs, medical wards, and medical-surgical wards from acute care hospitals from the 4 US geographic regions was conducted. Critical access hospitals, less than 6 months of BC data, and non-US hospitals were excluded. The study included BC use data from September 1, 2019, to August 31, 2021. Data were analyzed from February 23 to July 14, 2024.

**Main Outcomes and Measures:**

The primary outcome was BC use per 1000 patient-days. Adjusted means with 95% CIs were calculated using mixed-effects negative binomial regression models adjusted for unit type, hospital bed size, geographic region, seasonality, and state COVID-19 case load, with random intercepts accounting for clustering at unit and hospital levels. Secondary outcomes included blood culture positivity, single BCs, BC contamination, and minimum threshold for BC use where blood culture positivity would be optimized.

**Results:**

A total of 362 327 blood cultures were analyzed from 27 medical ICUs, 35 medical-surgical ICUs, 121 medical wards, and 109 medical-surgical wards from 48 hospitals in 19 states and the District of Columbia. The adjusted mean BC use per 1000 patient-days was 273.1 (95% CI, 270.2-275.9) for medical ICUs, 146.0 (95% CI, 144.5-147.5) for medical-surgical ICUs, 80.3 (95% CI, 79.8-80.7) for medical wards, and 65.1 for medical-surgical wards. Blood culture use was significantly higher across all 4 unit types in hospitals with more than 500 beds compared with 500 or less beds and in the West-Midwest compared with other regions. Single blood culture and positive blood culture rates were below 10% across all 4 unit types. Of the 292 units, 97% had a mean BC contamination rate within 3% of the recommended threshold, and 51% were within 1%. The minimum BC use thresholds (ie, BC use below this number may represent undertesting) were 120 BCs per 1000 patient-days for medical ICUs, 80 BCs per 1000 patient-days for medical-surgical ICUs, and 30 BCs per 1000 patient-days for medical-surgical wards.

**Conclusions and Relevance:**

The findings of this study suggest that blood culture positivity may help determine appropriate BC use for individual unit types.

## Key Points

**Question:**

What is the blood culture use rate of medical and medical-surgical intensive care units (ICUs) and wards in the US?

**Findings:**

In this cross-sectional study, data from 362 327 blood cultures collected in 292 units from 48 US hospitals between 2019 and 2021 were evaluated. The adjusted mean blood culture use per 1000 patient-days was 273.1 for medical ICUs, 146.0 for medical-surgical ICUs, 80.3 for medical wards, and 65.1 for medical-surgical wards.

**Meaning:**

The data from this study may help inform initiatives to reduce unnecessary blood culture use while maintaining an acceptable blood culture positivity target.

## Teaser

This cross-sectional study describes blood culture use in US hospital intensive care units and wards.

## Introduction

Blood cultures (BCs) are the reference standard to diagnose bloodstream infections; however, their use in clinical practice is often suboptimal, with a large portion of BCs collected inappropriately (eg, single BCs, inappropriate volume per bottle, and BC contaminants) or in patients with low risk of a bloodstream infection.^1,2,3,4,5,6^ These concerns point to the need for improved BC stewardship, which may include establishing a benchmark for BC use that allows individual institutions to identify opportunities to improve. Large-scale studies evaluating BC use rates among adult patients or BC quality indicators in the US have not been conducted. A recent multicenter study^7^ involving 14 pediatric intensive care units (ICUs) in the US reported their median BC use was 146 BCs per 1000 patient-days. For adult patients, wide variation in BC use rates has been reported between centers. For example, the median BC use rate across 223 German ICUs was 60 BCs per 1000 patient-days, much lower than the mean BC use range of 220 to 270 BCs per 1000 patient-days reported for a medical ICU at a university hospital in the US.^1,8^ These data indicate the need to evaluate BC use for specific clinical areas in larger cohorts. Adult general medicine is a main factor in BC use in hospitals.^6^ In this study, we aimed to examine BC use rates across medical ICUs, medical-surgical ICUs, medical wards, and medical-surgical wards in a multicenter cohort of US hospitals. Additionally, we describe BC positivity for pathogens, BC contamination, and single BC rates in these units and estimate a minimum threshold of BC use to detect bacteremia.

## Methods

### Study Participants and Procedures

We performed a retrospective cross-sectional evaluation of BC use in units classified per the National Healthcare Safety Network as medical or medical-surgical ICUs, medical wards, and medical-surgical wards in US hospitals between September 1, 2019, and August 31, 2021. Hospitals were recruited through the Centers for Disease Prevention and Control Prevention Epicenter Program (CDC-PEP) and Society for Healthcare Epidemiology of America (SHEA) Research Network. The CDC-PEP includes 11 institutions in the US that conduct collaborative innovative research to improve health care quality and patient safety with a focus on prevention of health care–associated infections and antimicrobial resistance.^9^ The SHEA Research Network is a consortium of more than 100 unique health care facilities in the US and abroad.^10^ Critical access hospitals, qualifying units with less than 6 months of BC data, and non-US hospitals were excluded. We aimed for a minimum of 30 hospitals and no more than 50. Participating hospitals signed a data use agreement with Johns Hopkins University and obtained institutional review board approval from all facilities before sharing deidentified BC data. This study was approved by the Johns Hopkins Medicine Institutional Review Board as quality improvement initiative not involving human participant research. This report followed the Strengthening the Reporting of Observational Studies in Epidemiology (STROBE) reporting guideline.^11^

### Blood Culture Data and Definitions

The following deidentified BCs data were collected: accession number, collection unit, collection date and time, BC result, and encounter identification. COVID-19 hospitalization rates at the state level were obtained from a dataset of the US Department of Health & Human Services^12^ on January 31, 2023.

A single BC was defined as only one BC set collected in a 24-hour period. A positive BC was defined as a BC growing a pathogen that was not a contaminant. Blood culture contamination was defined as a single BC in a 24-hour period positive for one or more skin commensals (eTable 1 in Supplement 1). If a single BC set was collected in 24 hours and was positive for a skin commensal, it was considered a BC contamination. Geographic regions were defined according to the US Census Bureau.^13^

### Statistical Analysis

Data analysis was performed from February 23 to July 14, 2024. We used descriptive statistics, such as proportions and medians (IQRs), to describe cohort characteristics. We calculated BC use pooled means (95% CIs) (BCs as numerator and patient-days as denominator) using a mixed-effects negative binomial regression model adjusted for unit type, hospital bed size, geographic region, seasonality, and state COVID-19 case load (number of confirmed and suspected COVID-19 cases in the state), with random intercepts accounting for clustering at the unit and hospital level. We conducted a sensitivity analysis in which we calculated the adjusted mean BC use excluding subsequent positive BCs for *Staphylococcus aureus* or *Candida* spp These pathogens may cause persistent bloodstream infection and appropriately lead to higher BC use as clinicians seek to document clearance of the bloodstream infection; thus, a higher prevalence of BCs positive for these pathogens might overestimate BC use. Additionally, we calculated the median (IQR) BC use per 1000 patient-days for each unit type (medical ICUs, medical-surgical ICUs, medical wards, and medical-surgical wards) using unit-quarter data stratified by hospital bed size (>500 or ≤500 inpatient beds) and region. We calculated adjusted means for single BCs, BC positivity, and BC contamination using mixed-effects generalized linear models adjusted for unit type, hospital bed size, region, seasonality, and state COVID-19 case load, with random intercepts accounting for clustering at the unit and hospital level.

The association between BC use and BC positivity was evaluated using unit-quarter data following the approach described by Karch and colleagues.^8^ First, we used a nonparametric regression followed by binomial general linear models (binomial family, logit link) with 2 linear segments representing 2 different regression slopes applied to distinct ranges of BC use to identify the low and top BC use range to detect true BC positivity. This minimum threshold definition holds the assumption that below this threshold an increase in BC use would result in increased BC positivity, but above this threshold no further increase in true positive BCs could be observed. Regression models were adjusted for variables identified a priori as likely to affect BC rates: unit type, hospital bed size, seasonality, geographic region, and COVID-19 case load per month at the state level. Unit quarters with BC use greater than 200 BCs per 1000 patient-days in medical wards and greater than 300 BCs per 1000 patient-days in medical-surgical wards were considered outliers and excluded from the model. All statistical tests were 2-sided, with *P* < .05 considered statistically significant. For comparison of means, unpaired tests were used. Data analysis was conducted using Stata, version 18.0 (StataCorp LLP).

## Results

### Cohort Characteristics

Forty-eight hospitals in 19 states and the District of Columbia provided data for 362 327 BCs from 27 medical ICUs, 35 medical-surgical ICUs, 121 medical wards, and 109 medical-surgical wards. Geographic distribution of participating hospitals included the South (54.2%), Northeast (20.8%), Midwest (18.8%), and West (6.3%) regions. The median bed size of participating hospitals was 321 (IQR, 208.0-660.5), and the median patient-days per month was 472.5 (IQR, 359-637) for medical ICUs, 369 (IQR, 254-501) for medical-surgical ICUs, 712 (IQR, 508-920) for medical wards, and 583 (IQR, 436-795) for medical-surgical wards. Table 1 summarizes the number of units participating by unit type, hospital bed size, and geographic region.

**Table 1.** Number of Participating Units by Hospital Bed Size and Geographic Region

Variable	No. (%)				
	All units (n = 292)	Medical ICU (n = 27)	Medical-surgical ICU (n = 35)	Medical ward (n = 121)	Medical-surgical ward (n = 109)
Bed size, No.					
≤500	133 (45.5)	7 (25.9)	25 (74.3)	30 (24.8)	71 (65.1)
>500	159 (54.5)	20 (74.1)	9 (25.7)	91 (75.2)	38 (34.9)
Geographic region					
Northeast	63 (21.6)	7 (25.9)	7 (20.0)	18 (14.9)	31 (28.4)
South	155 (53.1)	12 (44.4)	20 (57.1)	75 (62.0)	49 (45.0)
Midwest	52 (17.8)	5 (18.5)	5 (14.3)	19 (15.7)	22 (20.2)
West	22 (7.5)	3 (11.1)	3 (8.6)	9 (7.4)	7 (6.4)

Abbreviation: ICU, intensive care unit.

### BC Use Rates

**Figure 1.**
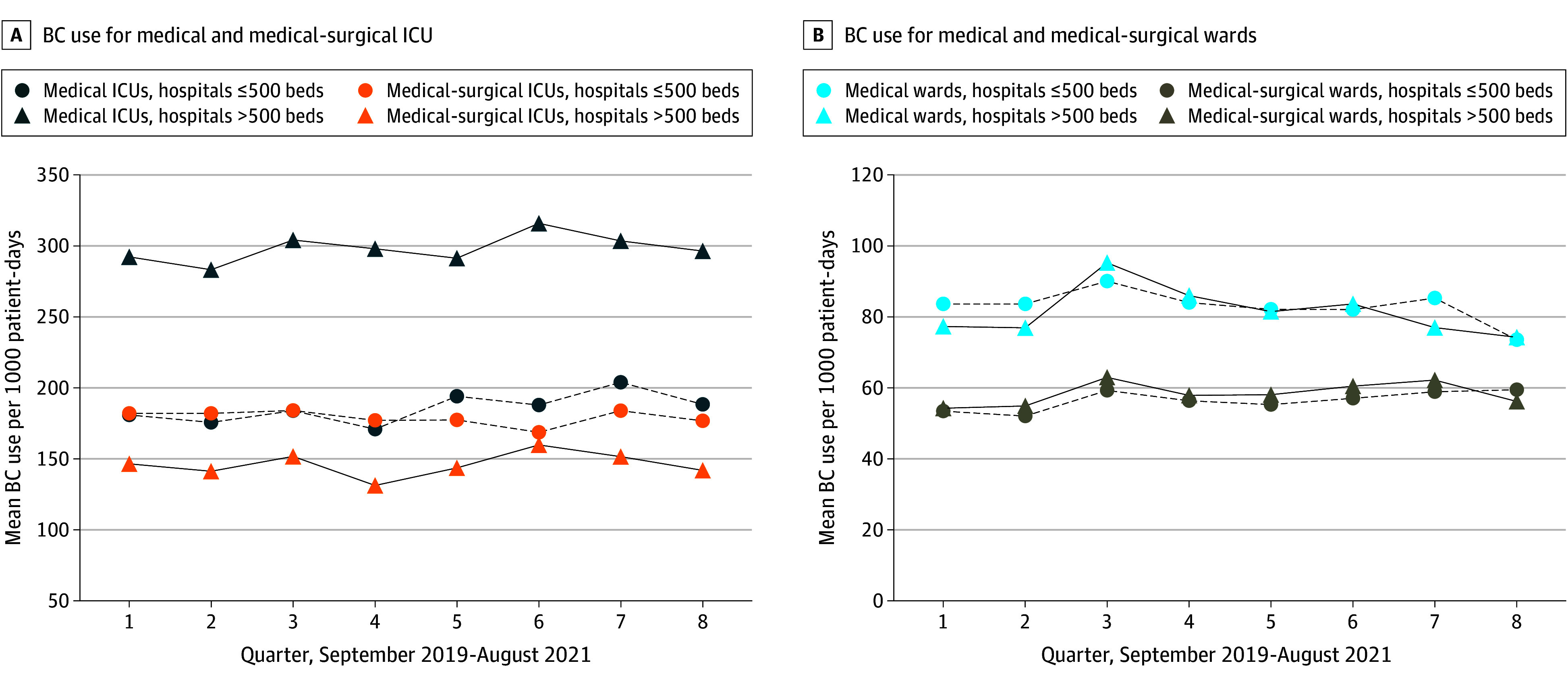
Quarterly Mean Blood Culture (BC) Use Per 1000 Patient-Days ICU indicates intensive care unit.

Adjusted BC use means were 273.1 (95% CI, 270.2-275.9) for medical ICUs, 146.0 (95% CI, 144.5-147.5) for medical-surgical ICUs, 80.3 (95% CI, 79.8-80.7) for medical wards, and 65.1 (95% CI, 64.8-65.5) for medical-surgical wards. Blood culture use was significantly higher across all 4 unit types in hospitals with more than 500 beds compared with 500 or less beds, and in the West-Midwest compared with the other regions (Figure 1 and Table 2). A sensitivity analysis excluding subsequent positive BCs for *S aureus* or *Candida* spp provided similar results (eTable 2 in Supplement 1). Median BC use per 1000 patient-days for each unit type stratified by hospital bed size is described in eTable 3 in Supplement 1.

**Table 2.** Adjusted Mean BC Use Per 1000 Patient-Days by Unit Type, Hospital Bed Size, and Geographic Region

Variable	No.		Adjusted mean BC use, (95% CI)
	Blood cultures	Patient days	
**Unit type**			
Medical ICU	83 062	299 981	273.1 (270.2-275.9)
Medical-surgical ICU	47 234	279 490	146.0 (144.5-147.5)
Medical ward	148 832	1 829 387	80.3 (79.8-80.7)
Medical-surgical ward	83 199	1 442 325	65.1 (64.8-65.5)
**Hospital bed size, No.**			
≤500			
Medical ICU	10 585	56 860	259.3 (255.3-263.2)
Medical-surgical ICU	33 417	184 780	141.4 (140.1-142.6)
Medical ward	38 428	464 134	75.4 (74.8-75.9)
Medical-surgical ward	47 223	824 356	62.9 (62.5-63.2)
>500			
Medical ICU	72 477	243 121	278.0 (274.7-281.2)
Medical-surgical ICU	13 817	94 710	159.1 (156.1-162.1)
Medical ward	110 404	1 365 253	82.0 (81.5-82.4)
Medical-surgical ward	35 976	617 969	68.9 (68.4-69.4)
**Geographic region**			
Northeast			
Medical ICU	17 624	64 445	275.2 (271.4-278.9)
Medical-surgical ICU	14 279	80 005	153.3 (151.1-155.5)
Medical ward	33 435	311 274	82.5 (81.8-83.2)
Medical-surgical ward	20 546	397 334	67.1 (66.7-67.6)
South			
Medical ICU	37 687	144 624	257.3 (255.1-259.6)
Medical-surgical ICU	22 718	136 773	137.5 (136.6-138.5)
Medical ward	76 136	1 078 044	76.4 (76.1-76.7)
Medical-surgical ward	33 255	566 095	60.7 (60.4-61.0)
West/Midwest			
Medical ICU	27 751	90 912	294.7 (290.4-299.0)
Medical-surgical ICU	10 237	62 712	160.1 (157.2-163.1)
Medical ward	39 261	440 069	88.6 (87.9-89.2)
Medical-surgical ward	29 398	478 896	69.9 (69.4-70.4)

Abbreviations: BC, blood culture; ICU, intensive care unit.

### BC Quality Indicators

There were no statistically significant differences in adjusted mean BC positivity: 6.5% (95% CI, 5.5%-7.6%) for medical ICUs, 6.2% (95% CI, 5.0%-7.3%) for medical-surgical ICUs, 7.3% (95% CI, 6.7%-7.9%) for medical wards, and 5.6% (95% CI, 5.0%-6.1%) for medical-surgical wards (Table 3). Eight percent of BCs were polymicrobial. The most common pathogen identified in positive BCs was *S aureus* (eTable 4 in Supplement 1).

**Table 3.** Adjusted Mean BC Positivity, Contamination, and Single BC Rates Using Unit-Quarter Data, August 2019-July 2021

Variable	Adjusted mean, % (95% CI)		
	BC positivity	BC contamination	Single BC
**Unit type**			
Medical ICU	6.5 (5.5-7.6)	1.50 (1.24-1.76)	6.3 (4.5 − 8.0)
Medical-surgical ICU	6.2 (5.0-7.3)	1.46 (1.18-1.75)	8.4 (6.5-10.3)
Medical ward	7.3 (6.7-7.9)	1.02 (0.89-1.14)	7.4 (6.1-8.7)
Medical-surgical ward	5.6 (5.0-6.1)	0.89 (0.76-1.02)	9.3 (8.0-10.6)
**Hospital bed size**			
≤500			
Medical ICU	5.8 (4.7-7.0)	1.60 (1.27-1.93)	8.9 (6.2-11.6)
Medical-surgical ICU	6.1 (4.9-7.2)	1.55 (1.23-1.87)	9.6 (7.4-11.8)
Medical ward	6.6 (5.8-7.5)	1.11 (0.91-1.31)	10.8 (8.4-13.1)
Medical-surgical ward	5.4 (4.8-6.0)	0.95 (0.80-1.09)	11.1 (9.3-12.8)
>500			
Medical ICU	6.8 (5.7-7.9)	1.46 (1.19-1.74)	5.4 (3.7-7.0)
Medical-surgical ICU	6.4 (5.2-7.5)	1.22 (0.95-1.49)	5.0 (3.6-6.5)
Medical ward	7.5 (6.8-8.2)	0.99 (0.86-1.11)	6.3 (5.1-7.5)
Medical-surgical ward	5.9 (5.2-6.6)	0.79 (0.64-0.94)	6.2 (5.1-7.4)
**Geographic region**			
Northeast			
Medical ICU	5.6 (4.5-6.7)	1.10 (0.85-1.36)	6.8 (4.7-8.9)
Medical-surgical ICU	5.4 (4.4-6.4)	1.00 (0.75-1.24)	7.2 (5.3-9.1)
Medical ward	6.1 (5.3-6.9)	0.73 (0.56-0.90)	7.5 (6.0-9.0)
Medical-surgical ward	4.9 (4.3-5.4)	0.63 (0.51-0.75)	8.8 (7.6-10.1)
South			
Medical ICU	7.0 (5.8-8.2)	1.49 (1.18-1.81)	7.7 (5.4-10.0)
Medical-surgical ICU	6.4 (5.2-7.7)	1.51 (1.17-1.84)	10.3 (7.9-12.8)
Medical ward	7.6 (6.8-8.4)	1.01 (0.86-1.15)	8.8 (7.1-10.6)
Medical-surgical ward	5.9 (5.2-6.7)	0.92 (0.76-1.08)	11.9 (9.7-14.1)
West/Midwest			
Medical ICU	6.6 (5.5-7.8)	1.86 (1.52-2.21)	3.7 (2.5-4.8)
Medical-surgical ICU	6.2 (5.0-7.4)	1.82 (1.46-2.18)	4.7 (3.4-5.9)
Medical ward	7.3 (6.5-8.0)	1.23 (1.05-1.40)	3.9 (3.1-4.8)
Medical-surgical ward	5.8 (5.0-6.6)	1.12 (0.92-1.32)	5.6 (4.5-6.6)

Abbreviations: BC, blood culture; ICU, intensive care unit.

Using all BCs as the denominator, the adjusted mean BC contamination rate was 1.50% (95% CI, 1.24%-1.76%) for medical ICUs, 1.46% (95% CI, 1.18%-1.75%) for medical-surgical ICUs, 1.02% (95% CI, 0.89%-1.14%) for medical wards, and 0.89% (95% CI, 0.76%-1.02%) for medical-surgical wards . The BC contamination rates using all BCs with organism growth as denominators are reported in eTable 5 in Supplement 1. Most units (97%) had BC contamination rates within 3%, and 51% of the rates were within the most recent recommended threshold of 1% or less (eTable 6 in Supplement 1). The most common organisms identified in BC contamination were coagulase-negative staphylococci (eTable 7 in Supplement 1).

The adjusted mean single BC rates were 6.3% (95% CI, 4.5%-8.0%) for medical ICUs, 8.4% (95% CI, 6.5%-10.3%) for medical-surgical ICUs, 7.4% (95% CI, 6.1%-8.7%) for medical wards, and 9.3% (95% CI, 8.0%-10.6%) for medical-surgical wards (Table 3). These differences were not statistically significant.

### BC Use Reference Range

**Figure 2.**
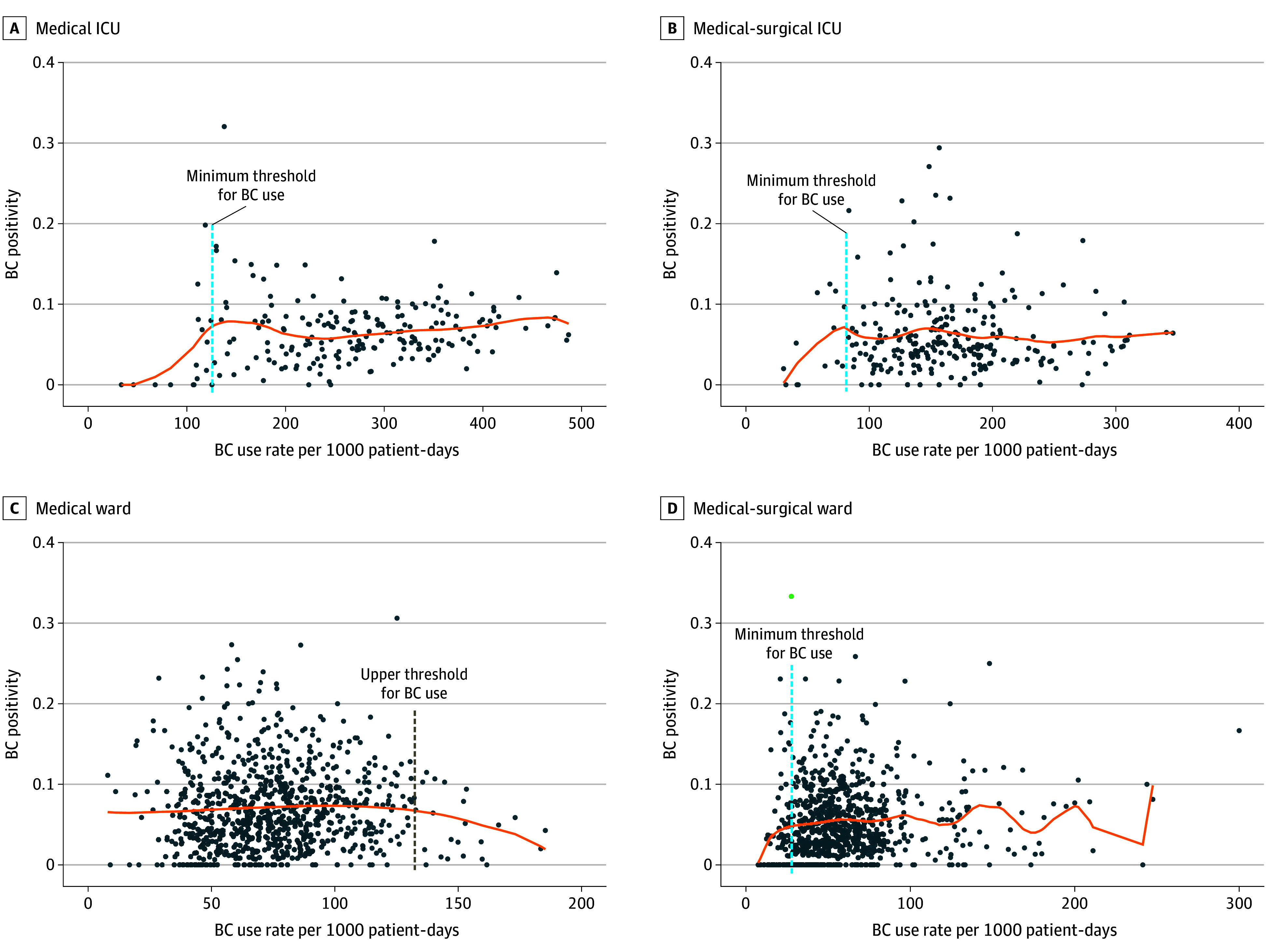
Association Between Blood Culture (BC) Use and BC Positivity Associations for medical intensive care units (A), medical-surgical ICUs (B), medical wards (C), and medical-surgical wards using nonparametric weighted regression based on the BC data without making any assumptions about the underlying distribution. Each dot in the x-axis represents a unit quarter. The y-axis represents BC positivity (10 increment). The orange line represents the conditional mean. Regression models were adjusted for unit type, hospital bed size, geographic region, seasonality, and monthly COVID-19 hospitalization rates at the state level.

We evaluated the association between BC use and BC positivity for each unit type to determine a BC use range using a regression model that accounted for hospital bed size, geographic region, seasonality, and COVID-19 hospitalizations at the state level. Based on the segmented regression model, the lower boundary of a BC use range was established at 120 BCs per 1000 patient-days for medical ICUs, 80 BCs per 1000 patient-days for medical-surgical ICUs, and 30 BCs per 1000 patient-days for medical-surgical wards (Figure 2). An upper boundary could not be established for these units, likely due to heterogeneity in patient population and local practices. Regression models did not identify a minimum BC use threshold for medical wards likely due to the narrow BC use range. However, the upper boundary was identified at 130 BCs per 1000 patient-days (BC use above this boundary was associated with a decrease in BC positivity). eTable 8 in Supplement 1 provides regression model output.

## Discussion

Blood cultures are the reference standard to diagnose bloodstream infections; however, most BCs ordered in routine practice do not grow organisms. Although the cause of negative BCs is multifactorial, overtesting (ie, testing of patients unlikely to have a bloodstream infection) and suboptimal collection practices (eg, collecting a single set rather than 2 sets) are major correctable contributors. To our knowledge, large-scale studies evaluating BC use and BC quality indicators in the US have not been previously conducted. In this multicenter study that included BC data from 292 adult medical ICUs, medical-surgical ICUs, medical wards, and medical-surgical wards from 48 hospitals located throughout the 4 US geographic regions between 2019 and 2021, we found BC use was associated with unit type, hospital bed size, and region. Blood cultures collected as single sets were uncommon; however, BC positivity was low.

Medicine services have higher BC rates than other services, such as surgery or oncology, and may account for 50% of hospital BCs.^1,6^ In our study, ICUs had higher BC use than general wards, and purely medicine units had higher use than their mixed medical-surgical counterparts. Furthermore, we estimated a minimum threshold for BC use (ie, BC use below this threshold represents undertesting) as 120 BCs per 1000 patient-days for medical ICUs, 80 BCs per 1000 patient-days for medical-surgical ICUs, and 30 BCs/1000 patient-days for medical-surgical wards. Our models were not able to establish an upper boundary for BC use for these units, likely due to variation in clinical practice across sites, sample size, and heterogeneity in patient population, despite evaluating this association among the same unit types. Clinicians’ main hesitation to engage in BC stewardship has been concern for missing an infection; therefore, having a minimum BC use threshold could be helpful.^14^ We had a larger sample of medical wards in this cohort than other unit types included, which allowed us to find an upper limit of BC use at 130 BCs per 1000 patient-days (ie, no further increase in BC positivity above this threshold); however, it did not find the lower value. This may be due to the narrower BC use range among medical wards. While these thresholds could be used as a reference, additional validation with a different dataset is warranted. Individual hospitals should track both BC use and BC positivity rates over time to understand assessments of opportunity and the effects of efforts to improve BC use and patient selection.

How BCs are collected (eg, number of BCs sets, use of both aerobic and anaerobic bottles) influences BC sensitivity.^15^ It has been estimated that 15% to 30% of positive BCs may be missed if blood samples are collected as single sets depending on the organism^16^ and a 10% threshold has been recommended by experts.^17,18^ In our study, 25% of the units were above this threshold looking at quarterly data. Blood culture use remains a cause of unnecessary antibiotic use and health care use.^4,5^ The Clinical and Laboratory Standards Institute considers laboratories should achieve BC contamination rates substantially below 3%, with less than or equal to 1% as the target rate.^19^ In this cohort, while most units were within the 3% target, only half met the more stringent recently proposed 1% threshold.

### Limitations

There are several limitations to our study. We did not collect patient-level data, such as diagnosis codes, and therefore could not risk-adjust BC use rates for case mix. We tried to mitigate this by generating BC use benchmarks for specific unit types. We acknowledge that methods for defining unit type in the National Healthcare Safety Network have limitations in application to varied practice settings. The study included times when practices may have been affected by the COVID-19 pandemic; however, BC use in our study was similar to that reported in studies conducted before the COVID-19 pandemic.^1^ Additionally, we accounted for COVID-19 hospitalizations in our regression models. While we included 48 hospitals from the 4 geographic regions in the US, we excluded critical access hospitals. Therefore, our findings may not be generalizable to very small hospitals. While we focused on clinical areas with high BC use volume, future research is needed to establish BC use benchmarks in other patient populations, such as the emergency department and oncology. We defined BC positivity and BC contamination electronically based on accepted definitions; however, reported BC contamination rates may be slightly different using other BC contamination definitions. Medical record review could help better adjudicate whether a positive BC is a false- or true-positive result; however, this would not have been feasible with 362 327 BCs.

## Conclusions

In this cross-sectional study of BC use in adult medical ICUs, medical-surgical ICUs, medical wards, and medical-surgical wards, we established crude benchmarks that institutions can use in their efforts to improve BC use. Due to variation in clinical practice and case mix that exists among hospitals, we suggest that BC use data should be tracked along with true BC positivity to better understand BC appropriateness and opportunities for improvement.

## Notes

Supplement 1. **eTable 1.** List of Organisms Included in the Blood Culture Contamination Definition **eTable 2.** Adjusted Mean Blood Culture Utilization (BCU) Rate per 1,000 Patient Days by Unit Type, Hospital Bed-Size and Geographic Region Excluding Subsequent Positive BCx for *Staphylococcus aureus* or *Candida* spp **eTable 3.** Median blood Culture (BCx) Utilization/1,000 Patient-Days, Single BCx, True BCx Positivity and BCx Contamination Using Unit-Quarter Data **eTable 4.** Most Common Pathogens Identified in Positive Blood Cultures **eTable 5.** Blood Culture Contamination (BCC) Using All BCx With Organism Growth as Denominator **eTable 6.** Proportion of Units Meeting the Old and New Recommended CLSI BCC Thresholds **eTable 7.** Most Common Organisms Identified in Blood Cultures That Met Bcx Contamination Criteria **eTable 8.** Segmented Regression Model to Develop an Optimal Blood Culture Utilization (BCU) Range

Supplement 2.
Data Sharing Statement
