# Supplementary material for: Blood Culture Use in Medical and Surgical Intensive Care Units and Wards
Source: JAMA Netw Open. 2025 Jan 15;8(1):e2454738. doi: 10.1001/jamanetworkopen.2024.54738 (PMC11736503; doi:10.1001/jamanetworkopen.2024.54738)
Supplement: Supplement 1. — eTable 1. List of Organisms Included in the Blood Culture Contamination Definition eTable 2. Adjusted Mean Blood Culture Utilization (BCU) Rate per 1,000 Patient Days by Unit Type, Hospital Bed-Size and Geographic Region Excluding Subsequent Positive BCx for Staphylococcus aureus or Candida spp eTable 3. Median blood Culture (BCx) Utilization/1,000 Patient-Days, Single BCx, True BCx Positivity and BCx Contamination Using Unit-Quarter Data eTable 4. Most Common Pathogens Identified in Positive Blood Cultures eTable 5. Blood Culture Contamination (BCC) Using All BCx With Organism Growth as Denominator eTable 6. Proportion of Units Meeting the Old and New Recommended CLSI BCC Thresholds eTable 7. Most Common Organisms Identified in Blood Cultures That Met Bcx Contamination Criteria eTable 8. Segmented Regression Model to Develop an Optimal Blood Culture Utilization (BCU) Range [file jamanetwopen-e2454738-s001.pdf]

Supplementary Online Content

Fabre V, Hsu YJ, Carroll KC, et al. Blood Culture use in medical and surgical intensive care units and wards. *JAMA Netw Open*. 2025;8(1):e2454738.
doi:10.1001/jamanetworkopen.2024.54738

eTable 1. List of Organisms Included in the Blood Culture Contamination Definition

eTable 2. Adjusted Mean Blood Culture Utilization (BCU) Rate per 1,000 Patient Days by Unit Type, Hospital Bed-Size and Geographic Region Excluding Subsequent Positive BCx for *Staphylococcus aureus* or *Candida* spp

eTable 3. Median blood Culture (BCx) Utilization/1,000 Patient-Days, Single BCx, True BCx Positivity and BCx Contamination Using Unit-Quarter Data

eTable 4. Most Common Pathogens Identified in Positive Blood Cultures

eTable 5. Blood Culture Contamination (BCC) Using All BCx With Organism Growth as Denominator

eTable 6. Proportion of Units Meeting the Old and New Recommended CLSI BCC Thresholds

eTable 7. Most Common Organisms Identified in Blood Cultures That Met Bcx Contamination Criteria

eTable 8. Segmented Regression Model to Develop an Optimal Blood Culture Utilization (BCU) Range

This supplementary material has been provided by the authors to give readers additional information about their work.

© 2025 Fabre V et al. *JAMA Network Open.*

eTable 1. List of organisms included in the blood culture contamination definition.

Coagulase-negative staphylococcus (see full list below)
Viridans group streptococci (see full list below)
Abiotrophia spp.
Aerococcus spp.
*Bacillus species**
Brevibacterium spp.
Corynebacterium species (exclude C. diphtheriae)
Cutibacterium (Propionibacterium) acnes
Dermabacter spp.
Granulicatella spp.
Kocuria spp.
Lysinibacillus spp.
Micrococcus species
Niallia (Bacillus) circulans
Paenibacillus spp.
Priestia (bacillus) megaterium
Rothia spp.
Ureibacillus (Lysinibacillus) massiliensis

***Excludes B. anthracis or thuringiensis, even if reported as cereus/thuringiensis.

List of viridans group streptococci and coagulase-negative staphylococci included in the blood culture contamination definition.

**Viridans group streptococci**	**Coagulase negative staphylococci**
S. alactolyticus	*S. arlettae*
S. anginosus	*S. auricularis*
S. australis	*S. capitis*
S. constellatus	*S. caprae*
S. criceti	*S. carnosus*
S. cristatus	*S. chromogenes*
S. downei	*S. cohnii*
S. gallolyticus	*S. condimenti*
S. gordonii	*S. epidermidis*
S. infantis	*S. equorum*
S. intermedius	*S. felis*
S. lactarius	*S. fleurettii*
S. massiliensis	*S. gallinarum*

© 2025 Fabre V et al. *JAMA Network Open.*

S. mitis	*S. haemolyticus*
S. mutans	*S. hominis*
S. oralis	*S. hyicus chromogenes*
S. parasanguinis	*S. kloosii*
S. peroris	*S. lentus*
S. pseudo pneumoniae	*S. muscae*
S. ratti	*S. nepalensis*
S. rubneri	*S. pasteuri*
S. salivarius group	*S. pettenkoferi*
S. sanguinis	*S. piscifermentans*
S. sinensis	*S. pulvereri*
S. sobrinus	*S. saccharolyticus*
S. vestibularis	*S. saprophyticus*
	S. sciuri
	S. simulans
	S. succinus
	S. vitulinus
	S. warneri
	S. xylosus

© 2025 Fabre V et al. *JAMA Network Open.*

eTable 2. Adjusted mean blood culture utilization (BCU) rate per 1,000 patient days by unit type, hospital bed-size and geographic region excluding subsequent positive BCx for *Staphylococcus aureus* or *Candida* spp. This sensitivity analysis was performed to avoid overestimation of BCU in hospitals that may have higher prevalence of *S. aureus* or *Candida* spp. cases which are situations in which repeat BC are indicated and infections more likely to lead to persistent infection than other organisms. We used a mixed effects negative binomial regression model that regress blood culture count on unit type, hospital bed size, region, seasonality, and number of state confirmed and suspected COVID cases with an offset of patient days and random intercepts accounting for clustering at unit and hospital level.

	**Blood cultures, N**	**Patient days, N**	**Adjusted BCU* Mean (95% CI)**
By unit type			
M-ICU	82,769	299,981	258.6 (254.69 - 262.54)
MS-ICU	48,982	279,490	141.2 (139.96 - 142.46)
M-ward	148,850	1,829,387	75.3 (74.72 - 75.83)
MS-ward	81461	1442325	62.8 (62.49 - 63.19)
By hospital bed-size			
≤500			
M-ICU	10,563	56,860	258.6 (254.69 - 262.54)
MS-ICU	35,169	184,780	141.2 (139.96 - 142.46)
M-ward	38,583	464,134	75.3 (74.72 - 75.83)
MS-ward	45385	824356	62.8 (62.49 - 63.19)
>500			
M-ICU	72,206	243,121	277.0 (273.78 - 280.30)
MS-ICU	13,813	94,710	158.8 (155.78 - 161.76)
M-ward	110,267	1,365,253	81.8 (81.32 - 82.26)
MS-ward	36076	617969	68.8 (68.26 - 69.26)
By geographic region			
Northeast			
M-ICU	17,496	64,445	274.3 (270.59 - 278.00)
MS-ICU	14,266	80,005	153.0 (150.86 - 155.19)
M-ward	33,268	311,274	82.3 (81.61 - 82.99)
MS-ward	20613	397334	67.0 (66.61 - 67.47)
South			
M-ICU	37,579	144,624	256.6 (254.37 - 258.79)
MS-ICU	24,488	136,773	137.4 (136.43 - 138.34)
M-ward	76,195	1,078,044	76.3 (75.99 - 76.55)
MS-ward	31484	566095	60.7 (60.38 - 60.96)
West/Midwest			

© 2025 Fabre V et al. *JAMA Network Open.*

M-ICU	27,694	90,912	293.7 (289.43 - 298.00)
MS-ICU	10,228	62,712	159.9 (156.93 - 162.79)
M-ward	39,387	440,069	88.4 (87.71 - 89.03)
MS-ward	29,364	478,896	69.8 (69.31 - 70.26)

M-ICU: medical intensive care unit, MS-ICU: medical-surgical intensive care unit, M-ward: medical ward, MS-ward: medical-surgical ward.

© 2025 Fabre V et al. *JAMA Network Open.*

eTable 3. Median blood culture (BCx) utilization/1,000 patient-days, single BCx, true BCx positivity and BCx contamination using unit-quarter data.

		**BCx utilization**			**Single blood culture**			**True BCx positivity**			**BCx contamination**		
	N	Median	Q1	Q3	Median	Q1	Q3	Median	Q1	Q3	Median	Q1	Q3
By unit type													
M-ICU	207	267.8	199.9	334.9	4.17%	2.27%	8.90%	6.21%	4.16%	8.38%	1.32%	0.82%	1.95%
MS-ICU	255	156.8	125.3	194.4	5.84%	2.86%	12.14%	5.05%	3.28%	7.80%	1.23%	0.34%	2.17%
M-ward	876	74.2	55.9	94.8	4.49%	2.38%	9.69%	6.37%	3.33%	10.01%	0.72%	0.00%	1.55%
MS-ward	804	53.9	39.9	72.3	5.44%	2.28%	13.56%	4.44%	2.09%	7.59%	0.00%	0.00%	1.47%
By hospital bed-size													
≤500													
M-ICU	54	174.8	124.4	222.5	6.34%	2.70%	10.17%	5.63%	2.08%	8.49%	1.42%	1.03%	2.29%
MS-ICU	188	156.8	126.6	198.6	7.85%	4.06%	13.38%	5.23%	3.33%	8.88%	1.56%	0.56%	2.39%
M-ward	228	66.4	49.1	101.1	6.08%	2.44%	11.68%	6.50%	2.85%	9.82%	0.72%	0.00%	1.62%
MS-ward	506	50.9	37.2	70.5	8.55%	3.57%	17.48%	3.79%	1.79%	7.14%	0.00%	0.00%	1.50%
>500													
M-ICU	153	297.7	246.3	352.8	3.78%	2.19%	7.16%	6.23%	4.42%	8.34%	1.31%	0.80%	1.83%
MS-ICU	67	155.7	123.2	190.9	3.08%	0.92%	5.99%	4.67%	2.62%	6.76%	0.62%	0.00%	1.32%

© 2025 Fabre V et al. *JAMA Network Open.*

M-ward	648	76.3	59.8	93.4	4.11%	2.34%	8.68%	6.25%	3.43%	10.21%	0.72%	0.00%	1.52%
MS-ward	298	58.8	45.4	77.4	2.95%	1.41%	6.11%	5.34%	2.69%	8.55%	0.00%	0.00%	1.39%
By geographic region													
Northeast													
M-ICU	55	271.1	167.1	336.7	9.39%	6.31%	13.23%	5.49%	4.09%	8.10%	1.13%	0.82%	1.64%
MS-ICU	56	172.0	125.8	219.4	5.44%	3.93%	10.44%	4.60%	2.87%	5.99%	0.49%	0.00%	1.23%
M-ward	134	84.8	63.7	132.8	7.04%	4.93%	10.08%	6.52%	3.77%	9.19%	0.85%	0.25%	1.62%
MS-ward	241	53.2	38.9	73.6	5.98%	3.08%	12.29%	3.42%	1.47%	6.56%	0.00%	0.00%	0.56%
South													
M-ICU	91	259.1	210.7	318.2	3.66%	2.17%	8.50%	5.74%	4.12%	8.11%	1.38%	0.60%	2.18%
MS-ICU	142	153.1	124.2	189.6	7.93%	2.83%	15.00%	5.88%	3.57%	9.38%	1.29%	0.34%	2.19%
M-ward	532	66.9	50.1	85.7	4.05%	2.38%	11.65%	6.16%	3.01%	10.63%	0.64%	0.00%	1.51%
MS-ward	347	53.3	40.1	70.9	8.82%	2.47%	21.05%	4.65%	2.13%	7.83%	0.00%	0.00%	1.47%
West/Midwest													
M-ICU	61	293.8	239.3	364.8	2.60%	1.65%	4.51%	7.05%	4.92%	8.48%	1.38%	1.04%	1.82%
MS-ICU	57	158.8	131.1	190.9	4.13%	2.11%	7.84%	4.26%	2.63%	6.29%	1.92%	1.00%	2.58%

© 2025 Fabre V et al. *JAMA Network Open.*

M-ward	210	86.2	70.6	104.7	3.62%	1.75%	6.32%	6.46%	3.77%	9.59%	0.82%	0.00%	1.67%
MS-ward	216	55.6	40.1	75.5	3.16%	1.67%	6.70%	5.39%	2.82%	8.36%	1.29%	0.00%	2.23%

M-ICU: medical intensive care unit, MS-ICU: medical-surgical intensive care unit, M-ward: medical ward, MS-ward: medical-surgical ward. Q: quartile

© 2025 Fabre V et al. *JAMA Network Open.*

eTable 4. Most common pathogens identified in positive blood cultures (excludes blood culture contaminants).

**Pathogen, N=27,198**	**Frequency**	**%**
Staphylococcus aureus	11,034	40.57
Escherichia coli	2,212	8.13
*Klebsiella spp.**	1,798	6.61
*Candida spp.***	1,730	6.36
Coagulase negative staphylococci, not *S. lugdunensis*	1,699	6.25
Enterococcus faecalis	1,671	6.14
Enterococcus faecium	1,061	3.90
Pseudomonas aeruginosa	815	3.00
Enterobacter species	455	1.67
Serratia marcescens	336	1.24
Viridans group streptococci	303	1.11
Proteus mirabilis	203	0.75
Bacteroides fragilis	163	0.59
Staphylococcus lugdunensis	154	0.57
Stenotrophomonas maltophilia	135	0.50
Lactobacillus species	128	0.47
Streptococcus agalactiae	125	0.46
Streptococcus pneumoniae	83	0.31
Morganella morganii	55	0.20
Cryptococcus neoformans	55	0.20
Group A streptococci	55	0.20
Granulicatella adiacens	48	0.18
Achromobacter xylosoxidans	47	0.17
Citrobacter koseri	42	0.15

**K. pneumoniae* (76.4%), % *K. oxytoca* (11.67). ***C. albicans* (35.37%), *C. glabrata* (26.82), *C. parapsilosis* (16.30%).

© 2025 Fabre V et al. *JAMA Network Open.*

eTable 5. Blood culture contamination (BCC) using all BCx with organism growth as denominator (rather than all BCx collected).

	BCC	No. of contaminants/ total no. of positive BCx
Overall	14.70%	4,261/28,996
By unit type		
M-ICU	17.93%	1,236/6,895
MS-ICU	18.60%	623/3,425
M-ward	11.95%	1547/12,950
MS-ward	14.58%	855/5,726
By hospital bed-size		
≤500		
M-ICU	21.10%	191/905
MS-ICU	20.41%	510/2,554
M-ward	13.21%	452/3,422
MS-ward	15.72%	487/2,982
>500		
M-ICU	17.45%	1,045/5,990
MS-ICU	12.97%	113/871
M-ward	11.49%	1,095/9,528
MS-ward	13.41%	368/2,744
By geographic region		
Northeast		
M-ICU	17.22%	232/1,347
MS-ICU	12.94%	99/765
M-ward	13.83%	404/2,922
MS-ward	9.03%	100/1,108
South		
M-ICU	19.09%	596/3,122
MS-ICU	18.38%	327/1,857

© 2025 Fabre V et al. *JAMA Network Open.*

M-ward	11.50%	765/6,654
MS-ward	11.86%	283/2,183
West/Midwest		
M-ICU	16.82%	408/2,426
MS-ICU	24.53%	197/803
M-ward	11.2%	378/3,374
MS-ward	19.4%	472/2,435

© 2025 Fabre V et al. *JAMA Network Open.*

eTable 6. Proportion of units meeting the old and new recommended CLSI BCC thresholds.

	1% threshold				3% threshold			
	>1%		<=1%		>3%		<=3%	
	n	%	n	%	n	%	n	%
CC:M	23	85.2	4	14.8	1	3.7	26	96.3
CC:MS	24	68.6	11	31.4	2	5.7	33	94.3
WARD:M	55	45.5	66	54.6	4	3.3	117	96.7
WARD:MS	42	38.5	67	61.5	2	1.8	107	98.2
Total	144	49.3	148	50.7	9	3.1	283	96.9

© 2025 Fabre V et al. *JAMA Network Open.*

eTable 7. Most common organisms identified in blood cultures that met BCx contamination criteria.

**Pathogen, n=4638**	**Frequency**	**%**
Coagulase negative staphylococcus, not *S. lugdunensis*	3,768	81.24
Viridans group streptococci	368	7.93
Micrococcus spp	134	2.89
Corynebacterium spp	115	2.48
Bacillus spp	108	2.33
Cutibacterium (Propionibacterium) acnes	52	1.12
Diphtheroids	34	0.73
Paenibacillus lautus	24	0.52
Rothia spp	20	0.43
Kocuria kristinae	5	0.11
Brevibacterium spp	6	0.13
Dermabacter spp	4	0.09

spp: species.

© 2025 Fabre V et al. *JAMA Network Open.*

eTable 8. Segmented regression model to develop an optimal blood culture utilization (BCU) range (below the threshold an increase in BCU results in increased BCx positivity, but above the threshold no further increase in true positive BCx could be observed). First, we generated dot-plots of BCU and true positivity. We then used nonparametric regression models to help identify the thresholds. We assumed that true positivity would increase as BCU increases up to a certain threshold. After reaching the minimum rate threshold, the increasing slope would become flat or less steep. Next, we used generalized linear models to analyze the association between BCU and true positivity using segmented approaches. Slopes were assumed differently before and after the thresholds. Adjusted odds ratios, 95% confidence intervals (CI), p-values, and Akaike Information Criterion (AIC) were used to determine the model specifications of the thresholds.

	**M-ICU**			**MS-ICU**			**M-ward**			**MS-ward**		
	AOR	95% CI	P value	AOR	95% CI	P value	AOR	95% CI	P value	AOR	95% CI	P value
BCU lower threshold	1.08	(1.06 - 1.11)	<0.001	1.02	(1.00 - 1.04)	0.080	1.00	(1.00 - 1.01)	0.280	1.03	(1.00 - 1.06)	0.042
BCU upper threshold	1.00	(1.00 - 1.00)	0.913	1.00	(1.00 - 1.00)	0.726	0.99	(0.98 - 1.00)	0.020	1.00	(1.00 - 1.00)	0.380
Hospital bed size												
<=500	1			1			1			1		
>500	0.98	(0.53 - 1.79)	0.936	0.86	(0.62 - 1.21)	0.392	1.09	(0.87 - 1.37)	0.456	1.33	(1.11 - 1.59)	0.002
Region												
Northeast	1			1			1			1		
South	0.99	(0.72 - 1.37)	0.955	1.40	(1.02 - 1.93)	0.038	1.27	(1.06 - 1.53)	0.010	1.26	(1.04 - 1.53)	0.020
West/Midwest	1.00	(0.68 - 1.47)	0.996	1.03	(0.58 - 1.82)	0.926	1.15	(0.96 - 1.36)	0.126	1.29	(1.05 - 1.58)	0.014
Season												

© 2025 Fabre V et al. *JAMA Network Open.*

Sept-Nov	1			1			1			1		
Dec-Feb	0.79	(0.66 - 0.94)	0.008	0.97	(0.82 - 1.14)	0.676	1.02	(0.91 - 1.14)	0.756	1.03	(0.87 - 1.22)	0.758
Mar-May	0.86	(0.71 - 1.04)	0.126	1.14	(0.87 - 1.48)	0.338	1.12	(0.99 - 1.27)	0.070	1.08	(0.92 - 1.26)	0.349
Jun-Aug	0.95	(0.77 - 1.17)	0.641	1.07	(0.84 - 1.37)	0.567	1.07	(0.95 - 1.21)	0.245	1.18	(1.01 - 1.37)	0.040
No. of hospitalized COVID cases	1.00	(1.00 - 1.00)	0.238	1.00	(1.00 - 1.00)	0.851	1.00	(1.00 - 1.00)	0.543	1.00	(1.00 - 1.00)	0.235
Constant	0.00	(0.00 - 0.00)	<0.001	0.01	(0.00 - 0.07)	<0.001	0.05	(0.04 - 0.07)	<0.001	0.02	(0.01 - 0.04)	<0.001

© 2025 Fabre V et al. *JAMA Network Open.*
