# Supplementary material for: Blood Culture Use in Medical and Surgical Intensive Care Units and Wards
Source: JAMA Netw Open. 2025 Jan 15;8(1):e2454738. doi: 10.1001/jamanetworkopen.2024.54738 (PMC11736503; doi:10.1001/jamanetworkopen.2024.54738)
Supplement: Supplement 2. — Data Sharing Statement [file jamanetwopen-e2454738-s002.pdf]

Data Sharing Statement

Fabre. Blood Culture Use in Medical and Surgical Intensive Care Units and Wards. *JAMA Netw Open*. Published January 15, 2025. doi:10.1001/jamanetworkopen.2024.54738

Data
Data available: No

Additional Information
Explanation for why data not available: The data that supports the findings of the study are available in manuscript and supplementary material.
